# Extrafollicular and other non-germinal center B cell responses

**DOI:** 10.1093/jimmun/vkaf336

**Published:** 2026-03-01

**Authors:** Stephanie C Eisenbarth, K Maude Ashby, Oluwagbemiga A Ojo, Adam Williams

**Affiliations:** Center for Human Immunobiology, Northwestern University Feinberg School of Medicine, Chicago, IL, United States; The Department Medicine, Division of Allergy and Immunology, Northwestern University Feinberg School of Medicine, Chicago, IL, United States; Center for Human Immunobiology, Northwestern University Feinberg School of Medicine, Chicago, IL, United States; The Department Medicine, Division of Allergy and Immunology, Northwestern University Feinberg School of Medicine, Chicago, IL, United States; Center for Human Immunobiology, Northwestern University Feinberg School of Medicine, Chicago, IL, United States; The Department Medicine, Division of Allergy and Immunology, Northwestern University Feinberg School of Medicine, Chicago, IL, United States; Center for Human Immunobiology, Northwestern University Feinberg School of Medicine, Chicago, IL, United States; The Department Medicine, Division of Allergy and Immunology, Northwestern University Feinberg School of Medicine, Chicago, IL, United States

**Keywords:** extrafollicular, germinal center, B cell, humoral immunity

## Abstract

B cells exist as different subsets shaped by developmental cues, the activation environment, and the magnitude and nature of immune stimulus; such heterogeneity is essential for tailored immune responses. From both mouse and human studies, the past decade has seen significant advances in our understanding of the wide variety of B cell activation pathways. However, the terms currently used to specify the type of B cell response have not kept up with this progress. Furthermore, a myopic focus on the germinal center (GC) response for the generation of protective or pathogenic antibodies has left other types of T cell-dependent but GC-independent antibody responses loosely categorized together as “extrafollicular (EF) responses” resulting in ambiguity with respect to cellular and molecular mechanisms. Based on the historical definition, an EF B cell response refers to an activation/differentiation path that is anatomically distinct from the GC and occurs outside of the B cell follicle. If anatomical location helps dictate functional outcomes, it is time to reconsider broad use of the term EF. Nomenclature is always imperfect, but a rethinking of current B cell nomenclature is warranted. Doing so could lead to a clearer understanding of different cellular pathways to humoral immunity and help reveal unanswered questions in B cell biology.

## Introduction

Antibody responses (humoral immunity) originate from multiple types of naive B cells. Mature naive B cells are divided into follicular B, marginal zone (MZ) B, and innate-like B1 cells. The primary focus of most research on humoral immunity is on CD4^+^ T cell dependent (T-dependent) responses that originate from naive follicular B cells. B cell activation begins with BCR (B cell receptor) triggering and cell proliferation. These activated B cells can differentiate into memory or effector populations. Effector B cells (also known as antibody secreting cells [ASCs]) are further subsetted into proliferating plasmablasts (PBs) or terminally differentiated, non-proliferating plasma cells (PCs), which can be either short- or long-lived (SLPC vs LLPC). Defining the exact molecular signals that drive each of these distinct B cell fates remain areas of active research.

**Figure 1.**
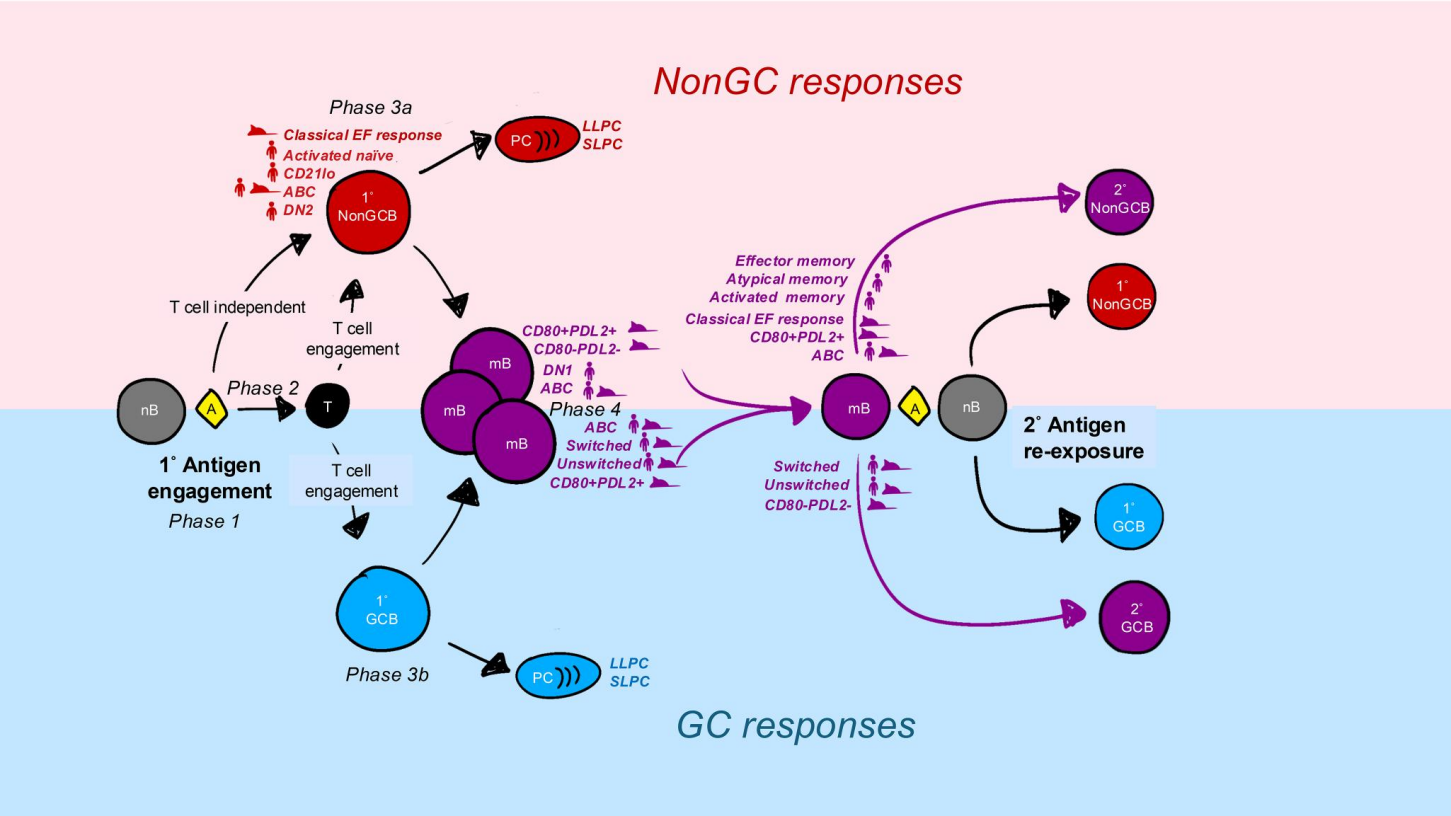
Currently used nomenclature of B cells with assumed or observed activation pathways. Around each B cell, terms that have been used to describe such cells are listed. Mouse-specific populations have a mouse symbol next to the name; human-specific populations have a human symbol. All B cells thought to derive from a non-GC response, including those considered extrafollicular, are colored red, whereas those thought to derive from a GC response are blue. Because the GC vs non-GC derivation of memory B cells are difficult to discern, they are colored purple. Abbreviations: A, antigen (yellow); T, CD4^+^ T cell (black); GCB, germinal center B cells (blue); mB, memory B cell (purple); nB, naive B cell (gray); LLPC, long-lived PC; PC, plasma cell; SLPC, short-lived PC.

This review will focus on one particular phase of B cell activation following initial licensing by T cells: the extrafollicular (EF) response, and other possibly related responses grouped together under the EF term (Fig. 1). EF responses were originally defined as the rapid appearance of proliferating B cell foci outside of follicles preceding germinal center (GC) formation during T-dependent responses, and as the sole outcome of T-independent B cell activation. The term EF is meant to contrast with GC responses, which occur within the follicles of secondary lymphoid organs (SLO). GCs are unique, well defined, anatomical regions composed of activated B cells that undergo specialized molecular processes including somatic hypermutation (SHM) and selection by T cells. Conversely, what is called “EF” has become anatomically uncertain and molecularly vague, inclusive of numerous B cell types, including ones that previously participated in GCs.

Here we review the original descriptions of EF responses in the context of a “textbook” T-dependent humoral response and then highlight how more recent research challenges this paradigm. We conclude by describing our approach to classifying different B cell responses, suggesting a more flexible and evolving nomenclature that incorporates recent insights to more precisely assign B cell activation sites. Why does such nomenclature matter? The immune system, composed of highly motile cells that traffic through diverse tissues and environments, has evolved spatial specificity. For B cells, as for all immune cells, location dictates functional outcomes because it determines the signals a cell experiences. Thus, identifying the spatially distinct paths to effective and long-lived antibodies could expand our approaches for vaccination and reveal targets to block pathogenic autoreactive, allogenic, or allergic antibodies.

## Why location matters: cellular anatomy of lymphoid organs

All lymphoid structures are spatially organized, whether it is a lymph node (LN), white pulp of the spleen, Peyer’s Patches (PP), thymus, or other lymphoid aggregates. This organization is reinforced through the ordered migration patterns of immune cells, instructed in part by chemokine gradients. Without such structure, rare antigen-specific T cells and B cells would be unlikely to find their cognate antigen or each other. The surveillance pattern of naive lymphocytes is therefore restricted to secondary lymphoid organs (SLOs). Within SLOs, concentrations of T cell zones and B cell zones exist, organized by chemo-attractant and chemo-repulsive gradients (eg CXCL13, oxysterols, S1P, CCL19, CCL21, etc).^1^^,^^2^ The B cell zones are classically called follicles; primary follicles are composed of mostly naive B cells whereas secondary follicles have GCs, which concentrate on-going B cell activation and selection with numerous support cells such as follicular dendritic cells (FDCs).

Many B cell subsets (eg follicular, MZ) and B cell states (GC vs EF) are named based on a location; however, B cells are not static and the actual activation sites are rarely studied. Where a B cell localizes within an SLO throughout various stages of humoral responses is crucial for functional differentiation as this determines the type of T cell help, exposure to differentiation cues such as cytokines, and antigen access.

Antigen drives B cell activation and selection following SHM through BCR stimulation and is provided to B cells through multiple paths. Naive B cells acquire small soluble antigens (<70 kDa) that pass directly from lymph into the B cell follicle likely through special conduits.^3–5^ In contrast, larger antigens (>7 0kDa) are excluded from the follicle and are trapped in the marginal sinus.^4–8^ Macrophages near the subcapsular sinus and dendritic cells (DCs) near high endothelial venules capture and display intact proteins to B cells in LNs.^9–12^ Antigen display by DCs to B cells occurs in non-follicular regions and primes B cells for interaction with CD4^+^ T cells.^13^ In addition, non-cognate B cells capture antigens in immune complexes from subcapsular sinus macrophages in a complement receptor (CR) dependent manner and deposit them on FDCs in the primary follicle prior to GC formation.^9^^,^^10^^,^^12^^,^^14–17^^,^^18^^,^^19^ Similar uptake and deposition of antigen on FDCs by non-cognate splenic marginal zone (MZ) B cells have also been described.^9^^,^^10^^,^^20^^,^^21^ Thus, antigen specific B cells initially become activated after BCR-mediated binding to cognate antigen displayed or transported by multiple cell types including primary follicle FDCs, non-cognate B cells, DCs, and macrophages.^14^^,^^22^

As the immune response progresses, antigen depots become increasingly rare in primary follicles and are predominantly thought to concentrate on GC FDCs.^22^ Specific antibody binding and clearance, antigen degradation as well as uptake by activated B cells likely contribute to the disappearance of antigen in the primary follicle. Collectively, these mechanisms facilitate the accumulation of tissue-derived antigen in lymphoid structures, allowing for rare antigen-specific lymphocytes to efficiently survey the entire body for their cognate antigen simply by trafficking between peripheral lymphoid organs. However, how antigen is preserved or displayed outside of GCs as the B cell response evolves is unclear and few studies have attempted to define the nature, site or persistence of antigen outside of the GC. One possibility is that non-GC B cells can traffic into the GC to sample antigen;^23^^,^^24^ however, such cells seem to remain within GCs rather than taking antigen out for a non-GC response. If mutation and selection continue following the first few days of B cell activation outside of a GC, especially with T cell help, where and how antigen is accessed is a crucial question, the answer may help clarify how EF and other non-GC responses are promoted.

## Phases of B cell activation following immunization or infection

Clearly defining the phases of B cell activation establishes an important foundation for describing different types of B cell activation. T-dependent B cell responses can be conceptually broken down into 4 phases^25^ following initial antigen encounter during a primary response (Fig. 1). The approximate timeline for each phase is indicated, but varies based on the nature of the immune challenge:

### Phase 1, initial antigen engagement (days 0–1)

Lymphocytes require at least two different signals during activation to avoid death or anergy: antigen and co-stimulation. Although both signals can be provided to T cells by dendritic cells, B cells get access to signal 1 and 2 in a more promiscuous way. Signal 1, BCR activation, comes from cognate antigen in follicles of SLOs. Here, encounter with soluble antigen that enters follicles via the conduit system can occur, but is more likely facilitated by import and display by other cells,^13^^,^^26^^,^^27^ especially for larger (>70 kDa), particulate, or opsonized antigens^9^^,^^10^ (see section 2 for more detail). The decoration of antigen by complement C3 fragments contributes to both signal 1 and 2 by facilitating the display of antigens on the surface of FDCs, and by bridging the complement receptor 2 (CD21) co-receptor complex to the BCR.^22^^,^^28–30^ Other innate immune triggers such as Toll-like receptor (TLR) ligands can provide Signal 2, as well as CD40L. Altogether, these signals prime a naive B cell for initial proliferation and directed migration within SLOs.

### Phase 2, initial T cell engagement and expansion (days 1–3)

Primed B cells increase chemoattractant receptors such as EBI2 and CCR7 to migrate to the follicle border. For T-dependent B cell responses, Phase 2 involves T cell help at the T–B border and interfollicular zone (IFZ). It is at this stage that BCR affinity impacts the ability of a B cell to survive into the next phase, as higher affinity BCRs increase the ability to receive T cell help and proliferate.^31^ Such licensed B cells can continue to proliferate at the perimeter of the follicle, distal from the T cell zone and adjacent to the marginal zone.^32^ At some point during Phase 2, CSR likely occurs to produce some non-IgM activated B cells.^33–35^ Then, at least 2 spatially, temporally, and molecularly separate fates await B cells during the next phase.

### Phase 3a, EF responses as originally defined (days 3–11)

Although the term extrafollicular had been used sporadically since the 1970s, the initial characterization of antigen-specific B cells responses outside of follicles were from Liu et al and Jacob et al, who identified antigen-specific B cell proliferation adjacent to the T cell zone and continuous with the bridging channel of the spleen in rats immunized with protein antigens in aluminum hydroxide.^36^^,^^37^ Note that neither paper explicitly called these responses EF. Many of these B cells had intracellular BCR staining and were therefore considered PBs and proposed to be contiguous with the PC response in the red pulp. Proliferating antigen-specific B cells were observed later in the response in GC follicles and called a follicular response. For primary T-dependent responses, these EF foci were small and subsided by the time a full GC formed; however, large EF foci formed during secondary responses.^37^ In contrast, for T-independent B cell responses, this was the major site for B cell proliferation and differentiation;^37^^,^^38^ however, more work is needed to define the spatiotemporal events that govern T-independent activation and will not be further discussed in this review. This early definition of follicular vs EF responses was thus based on the anatomical location of the two main foci of rapidly dividing B cell blasts in response to hapten-protein immunization and included class-switched B cells. Within the next few years, several papers used the term “extrafollicular B cell response” to refer to proliferative PB foci in mice at the outer T cell zone (likely the bridging channel) and red pulp of the spleen, and later, to PBs in the medullary cords of LNs.^35^^,^^39–44^ Thus, EF responses were defined originally by their location distal to the B cell follicle and the distinct process of rapid PB differentiation.

### Phase 3 b, GC response (>day 4)

Upregulation of particular chemoattractant receptors such as S1PR2 drives activated B cells and Tfh cells into the follicular interior, marking the beginning of the GC response.^45^ BCRs undergo SHM to produce progeny with variant sequences that may or may not recognize the original antigen. Higher affinity GC B cells are able to most efficiently capture and present antigen and thereby successfully compete for Tfh cell help, including CD40L and IL-21.^45^ With acute resolving infections or transient immunizations, this process often subsides within a few weeks after exposure due to a number of negative feedback mechanisms including T follicular regulatory cells, antigen-specific antibodies, and reduced antigen availability.^46^ However, persistent GCs have been observed in mice, humans, and monkeys during viral infections.^47–50^ Note that “follicular reaction” is often incorrectly used synonymously with a GC reaction, likely based on early descriptions of such responses as distinct from EF responses, but the follicle also includes B cells not participating in the GC, including naive B cells and possibly other activated B cells.^51^

### Phase 4, ASC and memory generation (continuous throughout reaction)

The outcome of a humoral immune response includes the generation of several distinct populations of antigen-specific cells, including circulating and tissue resident memory B cells and antibody producing LLPCs in bone marrow, spleen and lamina propria of the gut.^52^ The relative contribution of GC vs EF or other responses to these populations is not uniformly agreed upon. Such characterization is made difficult in part because memory B cell or ASC differentiation drives cells out of the GC and even the follicle itself, although many questions remain about this stage of differentiation.^45^ GC B cell differentiation into memory B cells is at least in part regulated by IL-4.^53^^,^^54^ However, from the earliest work on EF responses, it was clear that such responses also contributed to the memory B cell pool.^37^^,^^44^ Numerous recent studies have attempted to differentiate the memory populations derived from EF vs GC responses in mice. Notch2-dependent early memory B cells differ from later GC-derived memory B cells^55^ and a lack of CD80 and PDL2 markers have been used to identify memory derived from primary EF responses.^56^ Conversely, CD80^+^PDL2^+^ memory B cells had been inferred as GC derived;^57^^,^^58^ however, more recent work using fate mapping revealed that a significant proportion of these cells arise from outside of the GC,^56^ highlighting the shortcomings of using limited cell surface markers to infer anatomical origins.

Multiple different signals likely dictate whether an activated B cell in phase 3 will ultimately differentiate into an ASC. Whether BCR affinity impacts ASC differentiation from GCs^59–61^ or EF responses^40^^,^^62^ remains debated. Deletion of either IL-21 in T cells or IL-21 receptor on B cells leads to smaller GCs, an increase of memory-like B cells but impaired PCs.^63^^,^^64^ This suggests that IL-21 promotes GC-derived PCs; however, IL-21 also upregulates CD11c in mouse and human B cells,^65–67^ a marker of cells thought to originate from non-GC responses. Follicular stromal cells and myeloid cells also provide an environmental niche with signals for ASC output such as IL-6 and APRIL.^68^ It should be noted that proliferating ASCs (“PB Foci”) are likely always outside of a follicle regardless of GC vs non-GC origin, although this is rarely tracked and would require special labeling tools in mice to resolve. Therefore, use of the EF label at later stages of any immune response may conflate multiple different cellular mechanisms.

### Secondary responses: memory B cell reactivation

Experimental animal models permit separate analyses of primary vs secondary responses, whereas in human studies this often cannot be disentangled. From mouse studies, both primary and secondary responses induce EF responses as originally defined^37^ (Fig. 1). Both can also induce GCs, however, the contribution of GC-derived memory B cells to forming secondary GCs is debated, especially in the context of an evolving virus.^69^^,^^70^^,^^56^^,^^71–77^ Instead, B cells activated from the naïve pool dominate GCs during a secondary response,^50^ whereas memory B cells are poised to rapidly differentiate into ASCs outside of the follicles, in EF PB foci and subcapsular proliferative foci (SPF).^11^^,^^37^^,^^78^^,^^79^ Such a division could allow new B cells to mutate through a GC response to recognize viral variants, whereas memory B cells (possibly from an EF or GC primary response) provide humoral protection to the epitopes from the original infection.^72^ Thus, memory B cells derived from a GC during a primary response often engage in an EF response during secondary challenges, highlighting the importance of defining the phase of B cell activation when using such terms.

## Reconsidering the role of GC-independent responses in humoral immunity

Over the past few decades, the following model of T-dependent antibody production and B cell differentiation during an acute infection has become dogma, consistent with the phases described above: early (<7 d) B cell activation results in rapid low affinity memory B cells and short-lived IgM PBs in an EF response. Subsequently, higher affinity B cells are selected to enter a GC and there become class switched, high affinity memory B cells and PBs, and ultimately LLPCs that seed the bone marrow. Thus, the antibodies generated from the early EF responses “hold the line” until the higher affinity, class switched GC-derived antibodies clear the pathogen. This textbook model is based on a number of assumptions from indirect observations that have recently been challenged.

*Assumption 1, All mutated B cells are GC-derived.* The idea that SHM with selection can occur outside of GCs originally arose from studies of pathogenic B cell responses in autoimmune models in mice and when the GC structure or kinetics is disrupted by infection;^80–83^ in humans with autoimmunity, peripheral blood B cells presumed to originate outside of GCs (“activated naïve” and “DN2”)^84^ also have evidence of SHM.^85^ Data are emerging that this process may occur more often than originally appreciated. Using GC lineage tracing in immunologically intact mice, our recent work supports a model in which non-GC-derived IgA PCs are highly mutated and have evidence of AID-mediated SHM with positive BCR selection^86^ This is consistent with previous work in the gut showing the existence of B cells with a mutation rate greater than zero in PPs that lack a clonal relationship to adjacent GC clones, indirectly suggesting that SHM may not be strictly confined to the GC.^87^ And recent work has shown that CD11c^+^T-bet^+^ B cells accumulate substantially more mutations than naive B cells, despite not participating in the GC response during viral infection in mice.^88^ A phenotypically similar population has also been observed in humans following influenza or malaria vaccination.^79^^,^^89–91^ Conversely, recent work directly quantifying mutational load in PCs derived from GC-marked cells indicates that GCs can output both high affinity and low affinity PCs.^59^

*Assumption 2, Class switching happens in or depends on a GC.* Although the association between GC and CSR is often made, data long suggested —and a recent study directly demonstrated—that CSR happens *before* the GC phase^34^ and therefore cannot be GC-dependent. This does not mean that CSR *cannot* happen within a GC but rather that some or most does not. Original work in the IgA field showed that IgA CSR in PP occurred independent of GCs.^92^ Using systemic immunizations models in mice with adoptive transfers of BCR transgenic B cells, it was recently shown that non-GC responses produce a large fraction of memory B cells, including ones that are class switched.^93^ And using a method to label GC- vs non-GC-derived serum antibodies in mice following antigen immunization in alum, the early wave of antigen-specific IgG was not of GC origin.^72^ Indeed, infection or immunization models in the context of disrupted or genetically impaired GCs still produce numerous antibody isotypes.^80^^,^^94–97^ Importantly, if most or all CSR happens outside of a GC, then expression of AID is also not a faithful maker of GC participation, as AID is required for both CSR and SHM.

*Assumption 3, Instructive or selective signals in the GC are required to imprint longevity on a differentiating PC.* Although transcriptional studies have been done in an attempt to distinguish long- vs short-lived PCs, it still remains difficult to definitively identify each population, and the requisite signals needed to drive one versus the other during an immune response remain unclear. Using GC fate tracking in mice, recent work has shown that PCs derived from the GC vs pre-GC period have similar turnover rates in the bone marrow,^98^ indicating that the longevity of a PC is not dictated by participation in a GC. In fact, even PCs generated to T cell independent (and therefore GC independent) antigens can be long lived in the bone marrow.^99^^,^^100^

*Assumption 4, Protection during an acute primary infection relies on GC-derived antibodies.* Most viral infections are controlled or cleared before the peak of a GC has formed. Therefore, humoral protection during a primary infection must come from non-GC B cell responses, including EF-derived ones.^83^ GCs are essential for generating B cell mutants derived from the naive pool that can diversify when the non-GC response has failed to control the infection. GCs are also important for generating a high affinity memory B cell pool, which upon reinfection, rapidly differentiate into ASCs outside of GCs to provide prompt antibody production during a secondary infection. Therefore, non-GC responses are the principal method of quickly generating antibodies with sufficient affinity to control primary infections as well as generating secondary antibodies from memory B cell-derived ASCs during reinfection.

*Assumption 5, B cell responses that occur outside of spleen or LNs constitute an EF response.* In mouse and humans, a growing body of literature suggests that humoral responses can be induced in semi-organized structures outside of secondary lymphoid organs,^101^ such as tertiary lymphoid structures (TLS),^102^^,^^103^ solid tumors,^104^ the thymus,^105–107^ and in the perivascular space of inflamed tissues.^101^^,^^108^^,^^109^ These observations have prompted some authors to describe such responses as “extrafollicular”; however, this characterization ignores the heterogeneity of non-SLO B cell responses and the original process described as EF responses in the spleen. In some cases, humoral responses outside of secondary lymphoid organs appear to form genuine GCs with a proliferative dark zone, FDC-like cells and Tfh cells.^110–112^ In other cases, non-SLO responses occur independently of Tfh cells and without forming any discernable GC-like structure.^113^ Distinguishing these possibilities requires careful consideration, as both GC-like and non-GC-like responses have been reported in the same tissue, depending on the type and chronicity of the immune response. Furthermore, many tissue and TLS-associated B cells responses involve the re-activation of tissue-resident memory B cells,^113^^,^^114^ which may be derived from GC or non-GC responses, highlighting the possibility of complex differentiation pathways when memory B cells are activated outside of SLOs during a secondary response.

*Assumption 6, Antibody responses generated in the absence of GCs is an EF response.* In mice, the impact of non-GC-derived antibodies can be observed by genetically deleting the ability to form GCs—the nature of these GC-independent antibodies is often similar to those in GC-intact mice in terms of neutralizing capability, protective function, and pathologic functions. In our own work, we observed that deleting BCL6 in mouse T cells, resulting in loss of T follicular helper (Tfh) cells and GCs, only impairs a limited class of antibodies (IgG1 and IgE) without significantly impacting food- or toxin-specific IgA in the gut,^95^^,^^115^ or neutralizing IgGs to viruses or vaccines in the lung.^51^^,^^94^ Using a modestly “late” time point (day 60-90 post infection), we showed that these GC-independent antibodies were likely from LLPC.^94^ Others have similarly observed Tfh- or GC B-independent antibody responses to a wide variety of immunizations or alloimmunization.^96^^,^^116–118^ Recent work using LCMV infection in immunologically intact mice showed that GC-independent BCR mutation and selection can happen in parallel even when the GC is intact.^88^

Exactly how such GC-independent, class switched, high affinity, LLPCs are induced and selected remains unclear. Importantly, such observations raise questions about whether these various GC-independent responses are mechanistically equivalent to the previously described EF responses. This is especially true for responses generated in LNs rather than the spleen, where EF responses in the bridging channel and red pulp are clearly outside of the follicle. In the LN, whether B cell activation at the follicle edge including the T-B border qualify as EF remains debated; clarification of whether this constitutes a mechanistically similar B cell activation process as those originally described as EF including a rapid proliferative burst with ASC differentiation and then dissolution within a few days is crucial. After lung viral infection in wild type mice (ie with intact GCs) and GC-deficient mice, we observe class-switched B cells adjacent to activated T cells in the LN T-B border 2 wk after infection,^51^ which suggests a qualitatively distinct process than either a GC reaction or EF response. We next consider how the broad labeling of non-GC responses as “EF responses” may actually encompass several functionally distinct processes of B cell activation and how grouping them together could result in confusion.

## How current uses of an EF label might cause confusion

Currently, most uses of the EF label describe a B cell activation process in which the actual location is either unknown or assumed to be outside of a GC. For example, all of the following responses have been considered EF: an early IgM ASC response following infection or vaccination; B cell activation in a nonlymphoid tissue; a GC-derived memory B cell reactivated to differentiate into an ASC during a secondary response; a prolonged primary response at the T–B border; a prolonged primary response in a disorganized SLO; and a T-independent B cell response. Many of these responses are indeed outside of a follicle, while some are not, or are adjacent to the follicle. Lumping them together under one label could obscure possible mechanistic and anatomical differences between each of these responses. For example, SHM with T cell selection outside of a GC versus ASC proliferation foci likely require different environmental cues and supporting cells.

In addition, the description of all non-GC B-cell responses as “extrafollicular” is misleading because some of these responses may occur at the follicle border. Using *Blimp1* reporter mice to identify PCs, early stages of ASC differentiation can be identified at the T–B border,^119^ and such responses are often labeled EF. Using the framework of phases introduced earlier, this means that Phase 3a (EF response) is being used synonymously for phase 2 in some studies (ie same location and possibly stage of B cell differentiation) but not in others (ie distinct location and stage of B cell differentiation). These phases as originally described are distinct in location and certainly in the stage of B cell differentiation: phase 2 involves B cell interactions with T cells and selection resulting in B cell migration into a GC (phase 3 b) or away from the follicle, whereas phase 3a involves proliferation (with perhaps some T cell support of unclear signals) and ASC differentiation. If we do not distinguish between these 2 phases, we could miss important signals and pathways that regulate the fate of an antigen-specific B cell. Just as the coasts of any country are still part of that country, the follicle border is contiguous with the follicle, and therefore it would be confusing to consider the T–B border “extra” or outside of that region (ie extrafollicular). Instead, we favor use of a term to indicate a distinct process for ongoing (beyond day 5–7) B cell activation and selection at the follicle perimeter, such as “perimeter response” to describe those occurring at the T–B border and IFZ.

Another issue that has caused confusion is what to call memory B cell reactivation during a secondary response outside of the follicle. If most memory B cells (including ones derived from a primary GC response) do not participate in a GC response during a secondary reactivation, but instead proliferate and differentiate into ASCs outside of the follicles, should such a secondary response be considered EF? If not, what should reactivation of a GC-derived memory B cell outside of a GC be called? And more importantly, how would one actually know the origin of such an activated memory B cell?

The early EF descriptions following immunization with antigen in adjuvants clearly described a transient response (< 7 d) dominated by ASCs. However, chronic inflammatory conditions seen in autoimmunity or particular infections cause conditions that result in ongoing non-GC derived ASC formation, whose pathways may or may not be the same as those originally described as “EF”.^84^^,^^85^^,^^120^^,^^121^ B cells from such responses often display a set of markers or gene signatures (CD11c and T-bet in particular^79^) that have been referred to as “ABCs,” which can mean atypical, autoimmune-associated, age-associated, or activated B cell depending on the study.^122–127^^,^^128^^,^^129^ ABCs are most often observed in mice in contexts of strong innate stimuli or in autoimmune or old mice. In certain studies, mouse and human B cells with this phenotype respond poorly to BCR and CD40 stimulation and have been considered anergic, although this might have to do with how the BCR stimulation was performed.^120^^,^^122^

Although commonalities exist between ABCs described in different studies, phenotypic differences exist depending on the inflammatory context and therefore “ABC” does not refer to a single B cell subset.^130^ In some cases, cells with this phenotype are identified during a primary response, whereas in other studies they refer to a memory B cell population, especially ones poised to differentiated into ASCs.^90^^,^^120^ It is not clear how these varied B cell phenotypes are related to each other (Fig. 1). Further, although an EF derivation of ABCs is mostly assumed, it is possible that some types of ABCs are actually GC-derived.^131^ ABCs in humans are often identified by certain makers from peripheral blood B cells, and based on mouse work, assumed to be EF, but as discussed below this is conjecture.

In humans, it is almost impossible to know the phase 3 derivation of a B cell (ie GC vs non-GC), as it is difficult to track such responses in SLOs or to extrapolate from a particular B cell phenotype in the blood (Fig. 1). For example, human B cells lacking IgD (indicating they have been activated) but also lacking the memory B cell marker CD27 have been labeled “atypical” and are often assumed to arise from an EF response.^121^^,^^130^^,^^131^ Although CD27 has been a powerful marker to identify human memory B cells, it is not clear how the lack of CD27 defines a B cell as unusual,^132^ especially given more recent data finding such cells in healthy vaccinated adults.^91^ Defining a B cell population as “atypical” may be misleading our understanding of the biology.^132^ Note that CD27 cannot be used to define memory B cells in mice and therefore mechanistic studies are difficult. An alternative interpretation is that not all human memory B cells express CD27^120^^,^^131^ (just as not all human memory T cells express CD45RO). Similarly, expression of CD11c and T-bet expression, low SHM, or low CD27 and CD21 in memory B cell populations are often used to identify peripheral blood B cells that are considered EF derived.^130^ There is little evidence to directly support that the location or process that generated such B cells is outside a follicle.

## Conclusions, ideas, and many unanswered questions

Given the variety of B cells and conditions currently associated with EF responses, it is likely that distinct responses exist, each with possibly unique cellular mechanisms. If all non-GC responses are assumed to follow the EF pathway as originally defined, it is likely we are missing important cellular steps, interactions, and cues that guide low vs high affinity PC differentiation and short vs long-lived PC imprinting. Furthermore, an obvious limitation of the current term “EF response” is that it only infers location and not function (although even the particulars of the location referred to by EF is currently ambiguous). Under different conditions, PB foci and B cells undergoing SHM may reside in similar EF locations, however, it would be odd to name these different cellular responses the same thing. Similar to the DC subset field of the early 2010s, the B cell field needs to redefine what constitutes particular subsets and requires a new linguistic framework to clearly identify B cell states and activation pathways.^130^ This will continue to evolve as more data becomes available, if we are mindful that such differences could exist and certain cell labels currently in use might obscure rather than illuminate the underlying biology.

We propose that in a T-dependent response, it is possible that at least 3 fates (Table 1) await a recently activated B cell at the T–B border during phase 2 depending on: (a) the cumulative activation signals through the BCR (integrating effective antigen concentration and BCR affinity); (b) level of T cell help (number of activated T cells and nature of cytokines and co-stimulation); and (c) the presence of innate immune stimuli (pattern recognition receptor ligands, cytokines, and complement). In a polyclonal response these could all happen in parallel and are therefore in competition; thus, BCR affinities and antigen availability are relative between all the activated B cells and each response would likely have different kinetics. Furthermore, under certain conditions each of these fates can also be induced outside of secondary lymphoid organs.

**Table 1.** Hypothetical model of humoral immunity determinants.

Nature of stimulation	GC	EF	PR/Other non-GC
BCR signal strength	++	++	+
T cell help	++	+/−	+
Innate stimuli & Type 1 cytokines	+/−	++	+

BCR signal strength refers to the relative affinity of the BCR (B cell receptor) for antigen with respect to other competing B cells and the effective antigen concentration. Signals that drive different type of humoral responses. Abbreviations: EF, extrafollicular; GC, germinal center; PR, perimeter response.

Fate 1 —Extrafollicular response: in the presence of abundant antigen, and sufficient T cell stimulation, and moderately strong BCR affinity/activation and/or coactivation by innate immune stimuli, we predict that activated B cells would rapidly (first few days) differentiate into proliferating ASCs with minimal SHM but possibly following CSR in some cells. These cells would then migrate to the LN medulla or splenic bridging channel to continue proliferation and terminal ASC differentiation followed by exit to the periphery as memory B cells and PCs. The resultant humoral response would produce cells with low mutational loads but antibodies with moderate affinity.

Fate 2—Germinal center response: we predict that B cells with higher affinity BCRs, in the presence of a moderate effective antigen concentration that also receive strong T cell help would migrate into the follicle center and form GCs; here they undergo robust SHM and proliferation for weeks-months depending on the amount of antigen available and negative regulatory signals. The resultant humoral response would produce ASCs and memory B cells with high mutational loads and high affinity serological memory.

Fate 3—T-dependent non-GC responses: we speculate that lower affinity B cells in the presence of moderate effective antigen concentrations that receive adequate T cell help could potentially mutate and proliferate in a GC-independent manner for a limited period of time (weeks?). This would produce cells with modest mutational loads and moderate affinity antibodies. Depending on the Phase 3 location of such a response, we propose the label “perimeter response” (PR) to indicate an on-going cellular interaction between T and B cells at the T–B border. Many details about this third fate remain to be determined. What is the nature of the T cells that participate in such a response? What tissue microenvironments and support cells could enable B cell selection outside of the GC? What inflammatory signals drive non-GC but non-EF responses, and how do they contribute to humoral-based protection?

Numerous other questions emerge by slightly re-framing our textbook model of humoral immunity. Did SHM within the GC and SHM outside of a GC evolve for separate but complementary functions? What are the cellular mechanisms that support SHM outside of the GC? What signals (eg cytokines, innate immune receptor ligands, etc) and chemoattractant cues promote GC vs EF vs other non-GC responses? To add additional complexity, these signals and how B cells integrate them potentially changes with age.^133^ Although numerous cytokines, and Toll-like receptor ligands have been described that favor EF responses and chemokines necessary for organized GCs, a cohesive and comprehensive picture of different B cell activation pathways requires further research.

## Data Availability

No new data were created or analyzed in this study. Data sharing is not applicable to this article.
